# Achieving high precision in analog in-memory computing systems

**DOI:** 10.1038/s44335-025-00044-2

**Published:** 2026-01-07

**Authors:** Piergiulio Mannocci, Giacomo Larelli, Marco Bonomi, Daniele Ielmini

**Affiliations:** Dipartimento di Elettronica, Informazione e Bioingegneria, Politecnico di Milano, Milano, Italy

**Keywords:** Engineering, Mathematics and computing, Physics

## Abstract

Modern workloads challenge von Neumann architectures due to memory-processor data transfer. In-memory computing (IMC) enables in situ processing, with analog IMC (AIMC) based on resistive memories offering high-throughput and energy-efficient multiply-accumulate operations. Precision is limited by noise, device/circuit variations, and non-idealities. This work reviews error sources in AIMC and surveys mitigation strategies: bit slicing, residue number system, error correction codes, and mixed-precision iterative refinement, analyzing hardware implementations, overheads, and tradeoffs.

## Introduction

**Fig. 1:**
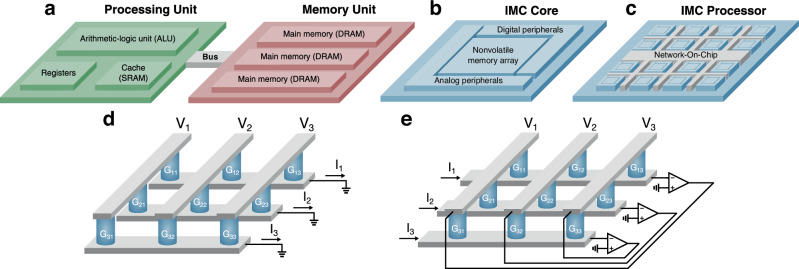
From von Neumann to in-memory computing. **a** Prototypical von Neumann architecture, where memory and processing units are physically separated and interconnected by a data bus. **b** IMC core, where computation happens directly within the embedded nonvolatile memory array. **c** IMC processor, composed of several IMC cores interconnected by a dedicated Network-On-Chip. **d** Open-loop IMC-based MVM circuit. **e** Closed-loop IMC-based IMVM circuit.

According to the von Neumann architecture (Fig. 1a), a computer consists of two essential parts, namely the central processing unit (CPU) and the memory unit^1^. The latter must support both instructions and data for the computation, which is executed in the CPU. With the massive increase of data and the widespread use of artificial intelligence (AI) in our modern digital society, memory and computing demands have seen an exponential increase^2^ for which the processing time and energy consumption are largely limited by the data movement between the memory and the CPU^3–5^. Overcoming this fundamental gap of performance requires the introduction of novel computing paradigms, leveraging unconventional physical mechanisms such as quantum effects or photonics.

Among the novel computing paradigms, in-memory computing (IMC) aims at executing data processing within the memory unit, thus alleviating the memory bottleneck^6,7^ (Fig. 1b). Various approaches and technologies have been proposed for IMC^8–10^. In general, IMC must rely on a precise, scalable, reliable, low-voltage/low-current memory device, which might be either a volatile or a nonvolatile memory technology. In addition to the conventional CMOS-based memories, such as static random-access memory (SRAM), dynamic random access memory (DRAM), and nonvolatile flash memory, various emerging memory technologies have been studied in the last 25 years. These NVM technologies include resistive switching memory (RRAM), phase change memory (PCM), spin-transfer torque (STT) magnetic random-access memory (MRAM), and ferroelectric random access memory (FeRAM)^11^. Volatile and nonvolatile memory technologies enable IMC with various architectures relying on either single-core IMC^12–16^ or multi-core IMC (Fig. 1c)^17–19^.

Analog IMC (AIMC) was shown to be particularly effective in accelerating data-intensive algebraic operations, such as the matrix-vector multiplication (MVM), when implemented using crossbar arrays (CBAs) of resistive memory devices. Figure 1d shows a conceptual scheme of an in-memory MVM accelerator, also known as open-loop AIMC, where a voltage vector **v** is applied on the columns of a CBA programmed with a conductance matrix **G**. Thanks to Ohm’s and Kirchhoff’s laws, acting as physical surrogates of multiply-accumulate (MAC) operations, a current vector **i** can be probed on the grounded CBA rows, namely:

1 $${\bf{i}}={\bf{G}}{\bf{v}},$$

corresponding to the MVM of matrix **G** and vector **v**. Thanks to the intrinsic parallelism provided by the CBA structure, the operation is performed in just one computational step or ${\mathcal{O}}(1)$, with a significant advantage over the typical ${\mathcal{O}}({N}^{2})$ computational complexity, where *N* is the matrix size, of MVM in digital processors. Open-loop AIMC has been experimentally demonstrated in a wide range of applications, including image processing^20,21^, neural networks^18,19,22^, and combinatorial optimization^23,24^.

The AIMC concept can be extended to inverse operations such as inverse-matrix-vector multiplication (IMVM) by using the closed-loop scheme illustrated in Fig. 1e^25–27^. The CBA is now enclosed in an analog feedback loop using on-chip operational amplifiers (OAs) in negative feedback configuration, which gives rise to virtual ground nodes at the CBA rows. By injecting a current vector **i**, the column voltages settle to match the same Ohm’s and Kirchhoff’s law of the MVM accelerator, i.e.:

2 $${\bf{v}}=-{{\bf{G}}}^{-1}{\bf{i}},$$

yielding the solution of the linear system described by the conductance matrix **G** and known term vector **i**. In the last few years, several closed-loop AIMC circuits have been proposed for a wide range of applications, including 5G and 6G communications^28–31^, ranking systems^32,33^, machine learning^34–36^, sensor fusion^30,37,38^, combinatorial optimization^39,40^ and general linear algebra^41–43^, demonstrating superior performance with respect to digital systems, which typically operate at ${\mathcal{O}}({N}^{3})$ complexity.

IMC has been generally applied to AI accelerators, where the MVM operation is used to accelerate each layer of a deep neural network (DNN), such as state-of-the-art convolutional neural networks (CNNs) for image recognition^18,44,45^. While DNNs are relatively robust with respect to errors and variations of IMC, other applications, such as scientific computing for quantum simulations^46,47^, computational biology^48^, genomics^49^, mechanical analysis^50^, as well as telecommunications^51^ and robot kinematics^52^, have limited tolerance with respect to computing inaccuracies. As a result, various types of mitigation techniques and architectures have been proposed to achieve high precision in limited-precision IMC systems. Table 1 summarizes the main mitigation schemes, including:

- slicing techniques, where the operands (coefficients and input values) are decomposed in multiple components in the digital (binary) or analog domain,
- residue number system (RNS), where the operands are decomposed as the modulo of chosen coprime numbers before carrying out multiplication and summation, finally reconstructing the results via the Chinese remainder theorem (CRT),
- error correction schemes, where a digital or analog error correction code (ECC) is adopted to detect and/or correct errors during IMC, and
- iterative refinement (IRF), where the computation results of a low-precision IMC unit are iteratively corrected by a higher precision unit in the so-called mixed-precision approach.

**Table 1:** Mitigation techniques for analog computing errors

Error source	Impacted block	Impacted operations	Mitigation technique
Quantization	Memory, Analog/digital and digital/analog converters	MVM, IMVM	Slicing, Residue number systems
Variability Noise Faults	Memory, Readout circuit, Analog/digital and digital/analog converters	MVM, IMVM	Error correction codes
Analog non-idealities	Array, Readout circuit, Analog/digital and digital/analog converters	IMVM	Iterative refinement

This work provides a detailed study of these schemes, providing a description and a comparison of various approaches in terms of accuracy and their tradeoffs with energy efficiency, area, and performance.

The rest of this work is organized as follows. Sec. “Error sources in AIMC systems” illustrates the main nonidealities and error sources of AIMC systems. Sec. “Analog and bit slicing” provides an overview of the slicing techniques, also known as *bit-width decomposition*, aimed at alleviating the impact of memory quantization. Sec. “Residue number system” describes RNS as a closely-related alternative approach to the slicing techniques for the mitigation of the requirements of converter precision. Sec. “Error correction codes” illustrates ECCs as a means to reject the impact of noise and variability on analog computing primitives. Sec. “Iterative refinement” addresses the IMVM-specific issues of circuit non-ideality and problem sensitivity by IRF. Finally, overheads and tradeoffs of the reviewed mitigation techniques are discussed in Sec. “Discussion”.

## Error sources in AIMC systems

**Fig. 2:**
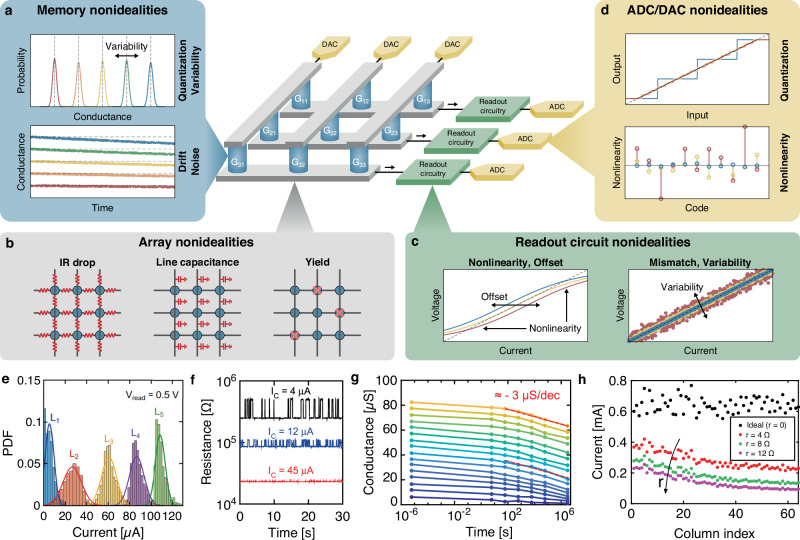
AIMC error sources. Overview of the main error sources impacting computation accuracy in AIMC, including (**a**) memory quantization, variability, and drift, (**b**) array-level IR drop, line capacitances, and device yield, (**c**) nonlinearity, offset, mismatches, and variability of readout circuits, and (**d**) nonlinearity and quantization of ADCs and DACs. Experimental data for (**e**) memory quantization, variability, and (**f**) noise in RRAMs, (**g**) conductance drift in PCMs, and (**h**) simulations of IR drop impact on current outputs of crosspoint arrays of resistive memory devices. **e** Reproduced with permission from^70^, licensed under a Creative Commons Attribution License. **f** Reproduced with permission from^59^. Copyright 2015 IEEE. **g** Reproduced with permission from^72^, licensed under a Creative Commons Attributions License. **h** Reproduced with permission from^63^. Copyright 2022 IEEE.

The lower latency and energy of AIMC come at the cost of reduced precision compared to the high precision of floating-point, von-Neumann-based digital processors^8,53^. Figure 2 highlights the main error sources in a typical AIMC primitive, including memory device and array parasitics, various nonidealities of the periphery circuit blocks, such as the analog/digital converter (ADC), digital/analog converter (DAC), as well as deterministic and stochastic variations of the readout circuitry. For instance, resistive memory devices typically suffer from quantization, i.e. limited bit precision^11,54,55^, programming variability^56,57^, i.e. deviation of the conductance from its nominal value at program time, drift, i.e. conductance increase or decrease over time^58^, and read variability and noise^59^ (Fig. 2a). Parasitic elements at the array level that typically affect the computing accuracy include the line resistance, which causes a voltage (IR) drop along columns and rows of memory arrays^60–64^, the line capacitance, which affects computation latency^65^, and device yield, which may result in unrecoverably stuck memory cells^66^ (Fig. 2b). Analog currents output by the memory array are typically preprocessed by dedicated readout circuitry before conversion, which may suffer from offset, variability, and nonlinearity^67^ (Fig. 2c). Circuit nonidealities and operational conditions play a significant role in closed-loop AIMC, where the OA finite gain and bandwidth contribute to the computing accuracy and latency, respectively, which are typically a function of the inverse problem sensitivity measured by the condition number *κ*^27,68^. Finally, ADCs and DACs inherently contribute to computation error owing to their limited bit-precision, differential and/or integral nonlinearity, and noise floor^69^ (Fig. 2d). Figs. 2e-h particularly report experimental data for nonidealities in resistive memory devices, including quantization and programming variability (Fig. 2e) and random telegraph noise (Fig. 2f) in resistive random access memories (RRAMs)^70,71^, conductance drift in phase change memories (PCM, Fig. 2g)^72^, and simulations of IR drop impact on crosspoint arrays of resistive memory devices (Fig. 2h)^63^. With respect to the ideal MVM of Eq. (1), the summation current in a real AIMC system is thus given by:

3 $$\tilde{{\bf{i}}}=({\bf{G}}+\delta {\bf{G}})\times ({\bf{v}}+\delta {\bf{v}})+\delta {\bf{i}},$$

where *δ***G,** *δ***v,** *δ***i** describe errors on the conductance matrix (e.g., memory and array nonidealities), input voltage vector (e.g., DAC nonidealities), and output current vector (e.g., readout circuitry and ADC nonidealities). Notably, errors may either be static, e.g., conductance mismatches arising from programming variability and IR drop or DAC offsets, or dynamically changing over time, e.g., as a result of read variability, conductance drift, and noise, thus requiring different compensation techniques. Similarly, with respect to the ideal IMVM of Eq. (2), the real IMVM operation performed by an AIMC system reads:

4 $$\tilde{{\bf{v}}}=-{({\bf{G}}+\delta {\bf{G}})}^{-1}\times ({\bf{i}}+\delta {\bf{i}})+\delta {\bf{v}}.$$

Several techniques have been proposed to mitigate the impact of device- and array-level nonidealities, including hardware-aware training of neural networks^62,73^, differential schemes for drift cancellation^58,72^, and mitigation schemes for IR drop^61,63,64^, allowing partial restoration of the nominal bit-precision of the memory cell.

## Analog and bit slicing

**Fig. 3:**
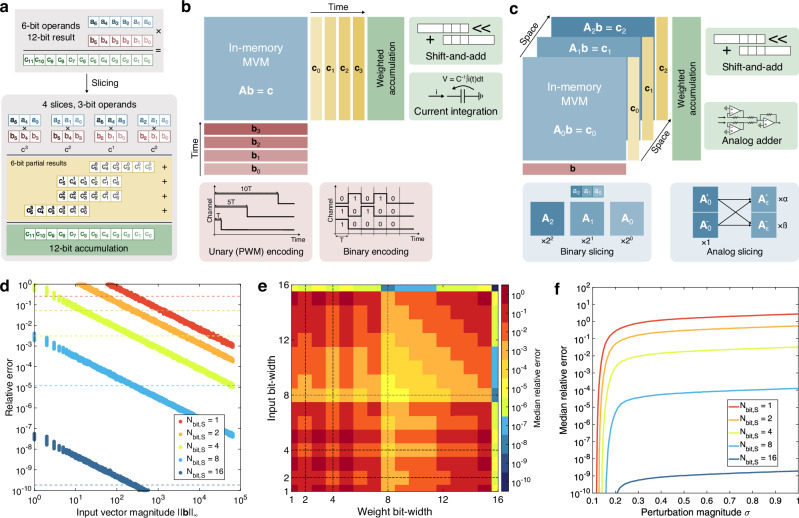
Slicing in AIMC. **a** Concept scheme of bit slicing technique for integer multiplication, where high-bit-width operands are decomposed in multiple low-bit-width slices. Partial results obtained by each sub-operation are shifted to reconstruct the equivalent significance of the processed slices and finally accumulated to reconstruct the target result. **b** Input vector slicing, where slices are typically processed sequentially by the same AIMC core. Different encoding schemes are used, including unary, also known as PWM, where values are represented by pulses of proportional duration and fixed amplitude, or binary encoding, where fixed-duration time slots are associated with different bit-significances. Each encoding must be matched to a conforming readout scheme for best performance, namely voltage-conversion followed by S&A for binary encoding and current integration-based schemes for PWM. **c** Owing to their stationarity, matrix slices are conversely encoded in space over different portions of the same memory array or different sub-arrays. Slice weights can be chosen as power-of-two multipliers, such as binary or quaternary encoding, or by dynamic computation at programming time in an analog fashion. In the first binary slicing approach, weight reconstruction is performed by digital-friendly S&A techniques. Conversely, the analog slicing approach is well-suited for reconstruction by analog adders with tunable gain. **d**–**f** Simulation results for a bit-sliced 128 × 128 MVM with 16-bit operands in a real IMC system for (**d**, **e**) *σ* = 0.1 LSB and (**f**) increasing values of *σ*. **d** Relative error as a function of the input vector magnitude for different values of input and weight slice bit-width. Dashed lines highlight the corresponding median relative error. **e** Median relative error as a function of the input and slice bit-width. **f** Median relative error as a function of *σ*.

Figure 3a shows a conceptual scheme of the slicing technique, which allows the processing of high bit-precision operations by computing modules with reduced bit-precision. Each operand **o** is represented by a linear combination of weighted *slices*, namely:

5 $${\bf{o}}={w}_{0}{{\bf{o}}}_{0}+{w}_{1}{{\bf{o}}}_{1}+\ldots +{w}_{k}{{\bf{o}}}_{k}=\mathop{\sum }\limits_{i=0}^{k}{w}_{i}{{\bf{o}}}_{i},$$

where **o**_*i*_, *w*_*i*_ are the *i*-th slice and slice weight, respectively, and the slicing basis {*w*_0_, *w*_1_, …, *w*_*k*_} is chosen such that each slice **o**_*i*_ can be represented using fewer bits than the original operand **o**. Under the slicing framework, the MVM operation between matrix **A** and vector **b** reads:

6 $${\bf{A}}{\bf{b}}=\mathop{\sum }\limits_{i=0}^{{k}_{1}}{w_{{\bf{A}},i}{\bf{A}}}_{i}\mathop{\sum }\limits_{j=0}^{{k}_{2}}{w_{{\bf{b}},j}{\bf{b}}}_{j}=\mathop{\sum }\limits_{i=0}^{{k}_{1}}\mathop{\sum }\limits_{j=0}^{{k}_{2}}{w_{{\bf{A}},i}w_{{\bf{b}},j}{\bf{A}}}_{i}{{\bf{b}}}_{j},$$

where each of the partial operations **A**_*i*_**b**_*j*_ is therefore an MVM between matrix **A**_*i*_ and vector **b**_*j*_ of reduced bit-precision. Among the most common choices for slicing bases are the *binary* and *unary* encoding, corresponding to bases {2^0^, 2^1^, …, 2^*k*^} and {1, 1, …, 1} respectively, owing to the relative ease of implementation of partial results accumulation. Binary encoding allows shift-and-add (S&A) to be used for implementing the weighted accumulation of slices. Conversely, digital counters can be used to efficiently combine partial results under a unary encoding.

Figure 3b shows a conceptual scheme of input slicing in an AIMC primitive, where input slices are processed sequentially in different time steps. Among the most common input slicing bases for AIMC are unary or pulse-width modulation (PWM) encoding, where different input values are represented by pulses of varying duration at fixed amplitude^13,74^, and binary encoding, where timeslots of the input waveform represent different bit-significances of the input word^75^, allowing reduction of DAC precision requirements to 1-bit^76^. To efficiently reconstruct the full-precision output, partial results provided by the AIMC macro must be accumulated in a weighted fashion by a conforming readout circuitry. In binary encoding schemes, partial current vectors are typically converted to voltage by transimpedance amplifiers (TIAs)^21,77^, digitized by ADCs, and accumulated by S&A. Conversely, PWM encoding is well-suited for accumulation by current integration-based converters, either realized by dedicated column capacitances^71^ or mixed-signal time-to-digital converters^13^.

A similar slicing can be applied to the MVM matrix as shown in Fig. 3c, where different slices are spatially mapped either to different columns or regions of a memory array^75,78^ or different memory arrays in a multi-core approach^21,79^. On the one hand, slice replication onto multiple cores enables the processing of different input slices in the same computational cycle^76,80^, albeit at the expense of increased hardware overhead. Conversely, spatially parallelizing the computation within the same core allows for a reduction in inter-tile communication overheads, at the cost of reduced array utilization efficiency. Common slicing bases for matrix elements include binary^75^ or larger bases such as quaternary encoding^76,80^, i.e. *w*_*k*_ = {4^0^, 4^1^, …, 4^*k*^}, by exploiting the multi-level cell (MLC) capability of resistive memory devices. Similar to binary slicing, quaternary encoding is compatible with S&A techniques, with two shifts being performed before each accumulation step. Fine-grained conductance tunability of memory devices^56,81^ can be further leveraged to allow using analog values for the slicing basis, with weighted accumulation performed by analog adders with tunable gain^21,79^. In this analog slicing approach, the slice weight is dynamically computed at program time by first calculating the error matrix between the target and programmed conductance matrices and subsequently rescaling the error matrix to fit the available conductance window of memory devices, allowing significant precision increase^21^ as well as mitigation of device variability^79^.

A major drawback of slicing techniques lies in *noise amplification*, namely the unwanted magnification of computing errors during the reconstruction process. Considering a real IMC system subject to nonidealities, the real MVM operation between matrix **A** and vector **b** reads:

7 $$\tilde{{\bf{c}}}={\bf{A}}{\bf{b}}+{\bf{n}}={\bf{c}}+{\bf{n}},$$

where ${\bf{n}} \sim {\mathcal{N}}(0,{\sigma }^{2})$ models random and deterministic errors detailed in Sec. “Error sources in AIMC systems”, Eq. (3). In the bit-slicing framework, a high-precision result is thus obtained by IMC-computed low-precision results as:

8 $$\tilde{{\bf{c}}}=\mathop{\sum }\limits_{i=0}^{k}{w}_{i}({{\bf{c}}}_{i}+{{\bf{n}}}_{i})\simeq {\bf{c}}+\mathop{\sum }\limits_{i=0}^{k}{w}_{i}{\bf{n}}={\bf{c}}+\alpha {\bf{n}},$$

where *α* is the *noise amplification factor*, such that the relative error reads:

9 $$\varepsilon =\frac{\parallel {\tilde{\bf{c}}-{\bf{c}}\parallel }}{\parallel {\bf{c}}\parallel }=\alpha \frac{\parallel {\bf{n}}\parallel }{\parallel {\bf{c}}\parallel }=\alpha \frac{\sigma \sqrt{N}}{\parallel {\bf{c}}\parallel} > \frac{\sigma \sqrt{N}}{\parallel {\bf{c}}\parallel },$$

where *N* is the matrix size. Figure 3d shows the relative error as a function of the input vector magnitude ∥**b**∥ for a bit-sliced 128 × 128 MVM with 16-bit operands **A** and **b**, highlighting a dependence on the slice bit-width *N*_*bit*,*S*_ owing to the different weighting factors *w*_*i*_. For example, considering 1-bit slices with weights {2^0^, 2^1^, …, 2^15^}, the overall amplification factor is equal to *α* = 2^16^ − 1. Conversely, by using wider 8-bit slices with weights {2^0^, 2^7^}, the amplification factor is reduced to *α* = 2^7^ + 1 ≪ 2^16^, thus resulting in significantly reduced relative error. The bit-widths of both the input and weight slices equally contribute to determining the noise amplification, as illustrated by Fig. 3e, although in a non-monotonic fashion. Conversely, the overall relative error exhibits a linear dependence on *σ* as shown in Fig. 3f.

## Residue number system

**Fig. 4:**
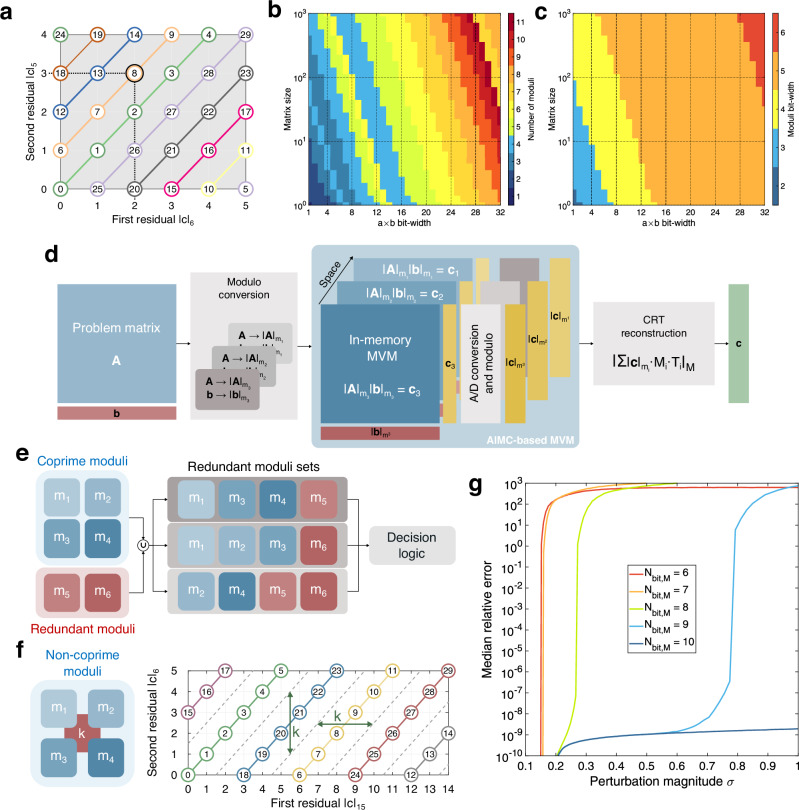
RNS-based AIMC architectures. **a** Concept scheme of RNS-based representation of integers {0, …, 29} using the moduli set {5,6}. Each integer is uniquely identified by a set of remainders with respect to the moduli set, with e.g., 8 corresponding to {2,3}. **b** Number of required moduli and (**c**) corresponding moduli bit-width as a function of the bit-width of partial multiplications *a*_*ij*_ × *b*_*j*_ and matrix size. **d** Concept scheme of in-memory RNS. Remainders of the problem matrix **A** and input vector **b** against a set of coprime moduli {*m*_1_, *m*_2_, *m*_3_} are first computed and forwarded to separate AIMC macros. Partial analog results are converted to the digital domain under the corresponding modulo. In the final reconstruction stage, the CRT identifies the unique number associated with the computed remainders. **e** The moduli set can be augmented with additional coprime moduli to create redundant moduli sets. Multiple candidate results are reconstructed by randomly grouping the available moduli and forwarded to a decision logic, typically implemented by majority voting, to mitigate the impact of burst-type errors. **f** Alternatively, non-coprime moduli sharing a common divisor *k* can be selected, allowing to tolerate small, distributed inaccuracies on the output of each modulo computation. **g** Median relative error for an RNS-based 128 × 128 MVM with 16-bit operands as a function of the perturbation magnitude *σ*, for increasing values of redundancy factor *k* from *k* = 1 (6-bit moduli) up to *k* = 16 (10-bit moduli), highlighting a nonlinear dependence on *σ*.

RNS is a number decomposition technique in which integers are represented by division remainders against a selected set of moduli^82^, i.e.:

10 $${\bf{o}}=\{| {\bf{o}}{| }_{{m}_{1}},| {\bf{o}}{| }_{{m}_{2}},\ldots ,| {\bf{o}}{| }_{{m}_{k}}\},$$

where *m*_*i*_ is the *i*-th integer of the moduli set, and ∣**o**∣_*m*_ (**o** modulo *m*) denotes the remainder of the integer division between elements of operand **o** and *m*, i.e.:

11 $$| {\bf{o}}{| }_{m}={\bf{o}}-m\times {\bf{n}},$$

where **n** is the vector of *folding integers*, such that for each element *o*_*j*_ of **o** it holds *o*_*j*_ < *m*. A number represented under the RNS framework can be reconstructed by leveraging the CRT or Sunzi Theorem^83^,

12 $${\bf{o}}={\left|\mathop{\sum }\limits_{i = 1}^{k}| {\bf{o}}{| }_{{m}_{i}}\cdot {M}_{i}\cdot {T}_{i}\right|}_{M},$$

where *M* = ∏_*i*_*m*_*i*_ is the *dynamic range*, *M*_*i*_ = *M*/*m*_*i*_, and *T*_*i*_ is the multiplicative inverse of *M*_*i*_ under the *m*_*i*_ modulo, i.e. $| {M}_{i}{T}_{i}{| }_{{m}_{i}}=1$. When all *m*_*i*_ are chosen to be pairwise coprime, each number in the range [0, *M*) is uniquely identified by a set of remainders, thus guaranteeing an exact reconstruction. Figure 4a schematically illustrates the RNS representation of numbers from 0 to 29 using moduli {6,5}, where each number is uniquely associated to a remainder pair, e.g., 8 corresponds to {2,3}.

RNS can be used to decompose operations on high bit-precision operands into partial operations on operands with reduced bit-precision. For an *n*-moduli set, the MVM between a weight matrix **A** and a vector **b** is represented under RNS as:

13 $${\bf{A}}{\bf{b}}=\{| {\bf{A}}{\bf{b}}{| }_{{m}_{1}},| {\bf{A}}{\bf{b}}{| }_{{m}_{2}},\ldots ,| {\bf{A}}{\bf{b}}{| }_{{m}_{n}}\}.$$

Thanks to the compatibility of modulo operation with addition and multiplication, each partial operation can be rewritten as:

14 $$| {\bf{A}}{\bf{b}}{| }_{{m}_{i}}={\left\vert | {\bf{A}}{| }_{{m}_{i}}| {\bf{b}}{| }_{{m}_{i}}\right\vert }_{{m}_{i}},$$

which reduces the required bit-precision to ${\log }_{2}({m}_{i})$ for memory and input values while simultaneously allowing to obtain computation results at ${\log }_{2}(M) > {\log }_{2}({m}_{i})$ precision. Residuals provided by the low-precision moduli are combined to identify the unique reconstruction candidate in the ${{\mathbb{N}}}^{k}$ space spanned by the set of moduli^84^. Fig. 4b shows the number of required moduli while Fig. 4c shows their corresponding bit-width as a function of the matrix size and of the overall bit-width of partial multiplications *a*_*ij*_ × *b*_*j*_, where *a*_*ij*_ and *b*_*j*_ are elements of the matrix **A** and vector **b** respectively. Figure 4d shows a conceptual scheme of an AIMC-based RNS implementation^85^, where partial operations are performed by AIMC cores operating at reduced bit-precision, whereas reconstruction is performed in the digital domain by CRT.

RNS has been widely explored for implementing power-efficient digital signal processors (DSPs) in embedded systems^86–89^, as well as for accelerating DNN inference in fully-digital^90–92^ and GPU-based systems^93^. More recently, RNS has been proposed for digital near-memory^94^ and in-memory oriented architectures^95,96^, with core MAC operations realized by digital logic. Demirkiran et al.^85^ first explored the possibility of implementing RNS in AIMC systems, with specific application to photonic MVM accelerators for DNN training^97^. With respect to slicing techniques, RNS has the compelling feature of reducing the required precision of output ADC in AIMC systems to the same precision of the memory devices and DACs^85^.

On the other hand, RNS suffers from high sensitivity to computational errors, owing to the large amplification applied to the residuals during reconstruction by CRT^84^, similar to noise amplification in bit slicing. Various techniques have been proposed to improve RNS robustness by leveraging information redundancy. Watson and Hastings^98^ proposed to augment the moduli set with additional redundant moduli, as shown in Fig. 4e. Partial results from different moduli can then be grouped into several distinct sets of moduli, each providing a potential reconstruction via CRT. Candidate solutions are then selected by a suitable decision logic such as majority voting^85,99,100^, typically allowing rejection of inaccurate results from few faulty units with large, burst-type errors.

A more suitable alternative for the inaccuracy scenario in AIMC, where IMC cores are typically characterized by small, distributed errors, is represented by the modulo-level redundancy proposed by Xiao et al.^84^. By lifting the requirement for moduli in the selected set to be pairwise coprime, common divisors provide redundant information implicitly shared across the different moduli, enabling both accurate and approximate reconstruction by identifying the nearest valid remainder tuple following geometrical considerations. The added complexity of the reconstruction process is entirely handled by the digital logic, while the AIMC cores continue to operate on moduli of slightly larger bit-width due to the common divisor. Fig. 4f schematically illustrates the added redundancy for a RNS system with moduli {15,6}, sharing a common divisor *k* = 3. With respect to the {5,6} RNS system of Fig. 4a, the redundant system can tolerate inaccuracies up to ±*k*/4 = ± 0.75 in the computation of residuals without incurring in reconstruction error. Figure 4g shows the beneficial impact of non-coprime moduli in terms of the median relative error as a function of the AIMC system perturbation magnitude *σ*, for an RNS-based 128 × 128 MVM between 16-bit operands, for an RNS system operating with 6-bit moduli and zero redundancy, and redundant RNS systems with increasing common divisors *k* = 2, 4, 8, 16, corresponding to 7-bit, 8-bit, 9-bit, and 10-bit moduli respectively. Note that RNS systems typically exhibit abrupt transitions between optimal low-error regions and high-error regimes, in stark contrast with the linear dependence on *σ* observed in the bit-slicing framework, owing to the nonlinear reconstruction process exploiting modulo-based operations in place of linear S&A.

## Error correction codes

**Fig. 5:**
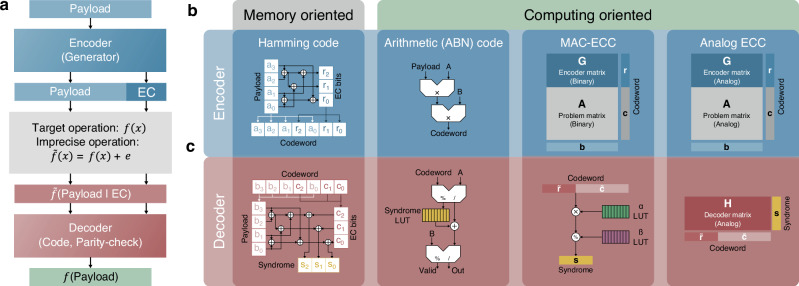
Error correction code system. **a** Concept scheme of an ECC system, where an encoder generates EC bits which are appended to the to-be-protected payload to form a codeword. Inaccuracies in the target operation result in a corrupted codeword, which is forwarded to a decoder exploiting the redundancy carried by EC bits to detect and correct errors, yielding the accurate computation output. **b** Encoder and (**c**) decoder schemes for AIMC, including memory-oriented Hamming codes for bit-level protection, and computing-oriented approaches, namely arithmetic ABN codes, MAC-ECC, and analog ECC, for protection of MVM outputs.

ECCs were first proposed in 1950 by Hamming^101^ as a way of controlling errors in data transmission over unreliable communication channels. Figure 5a shows a conceptual scheme of an ECC system where a payload *p* containing sensitive data is first processed by an *encoder*, which computes *r* error correction (EC) bits. The *codeword* formed by the payload data and the EC bits is processed by the target operation *f*(*x*), ideally providing an exact result *f*(*p*∣*r*). Different sources of inaccuracy, such as computation error, noise, or transmission faults, typically result in an inaccurate operation $\tilde{f}(x)=f(x)+e$ being performed in place of *f*(*x*), such that the obtained output is $\tilde{f}(p| r)=f(p| r)+e$. A *decoder* block processes the inaccurate output codeword, leveraging the redundant information carried by the EC bits to detect and, eventually, correct errors, thus allowing the extraction of the ideal result of the computation, namely *f*(*p*)^102^. Several different ECC variants have been proposed, mainly divided into fixed-size block codes and arbitrary-length convolutional codes^103^, providing single-bit^101^ and multi-bit^104–106^ correction capability.

Figures 5b, c highlight the main (b) encoder and corresponding (c) decoder structures proposed in the latest years to realize AIMC-specific ECCs. Conventional *memory-oriented ECC* schemes typically used in DRAM can be employed to protect resistive memories at the level of individual bits^107,108^, where the required encoding and decoding logic can be realized either in the digital domain by XOR gates^107^ or using fully in-memory approaches via stateful logic gates based on resistive memory devices^108^. On the other hand, *computing-oriented ECCs* directly operate on the MVM output, providing higher compatibility with the AIMC framework^109–114^. Feinberg et al.^109^ first proposed an AIMC-specific implementation of AN and ABN codes, relying on modular computation and divisibility properties to detect and correct errors at the computing level. In AN codes, input data are digitally multiplied by a known integer *A* to form the codeword, which is then forwarded to a linear operation such as MVM. ABN codes further provide correction capability by including a second multiplier *B* and a more complex decoder scheme^109^ providing additive corrections to the decoded codeword. Li et al.^110^ introduced MAC-ECC, a computing-oriented scheme based on modified Berlekamp codes for the protection of partial sums on binary data matrices, exploiting a modulo-based digital decoder to ascertain computation validity and perform error detection. To reduce the associated digital logic overhead and allow protection of continuously-valued analog conductances, Roth et al.^111,112^ introduced analog ECC relying on MVM-based encoder/decoder pairs, which can be embedded in the AIMC primitive with reduced area overhead. Li et al.^66^ experimentally demonstrated the effectiveness of analog ECC for single-error correction in the event of noise injection and stuck-on/off devices, with ECC-based primitives achieving nominal accuracy on the MNIST classification task. Multiple-error-correcting codes have also been proposed^114^ to extend further the capability of analog ECC for AIMC cores, enabling resilience from multiple faulty devices and error sources, typically at the cost of increased redundancy^113^.

## Iterative refinement

Inverse problems arise naturally in many applications, including machine learning^26^, telecommunications^28^, autonomous vehicles^30^, genomics^49^, and robotics^115^. However, their numerical solution shows limited robustness to inaccuracies owing to the magnification of algebraic errors^116^. The sensitivity of an inverse problem **Ax** = **b** to perturbations in both the coefficient matrix **A** or constant vector **b** is typically controlled by the condition number *κ*, namely the ratio of the maximal and minimal singular values *σ*_1_, *σ*_*n*_ of **A,** i.e. *κ* = *σ*_1_/*σ*_*n*_. As a common rule of thumb, up to ${\log }_{10}(\kappa )$ digits of precision are lost if no mitigation techniques are adopted^116^. To improve the solution accuracy of inverse problems, iterative refinement (IRF) was first proposed by Wilkinson in 1963^117,118^ in the context of linear systems, and subsequently extended to more complex inverse computations such as linear regression and general least squares^119,120^ and matrix decomposition^121^.

**Fig. 6:**
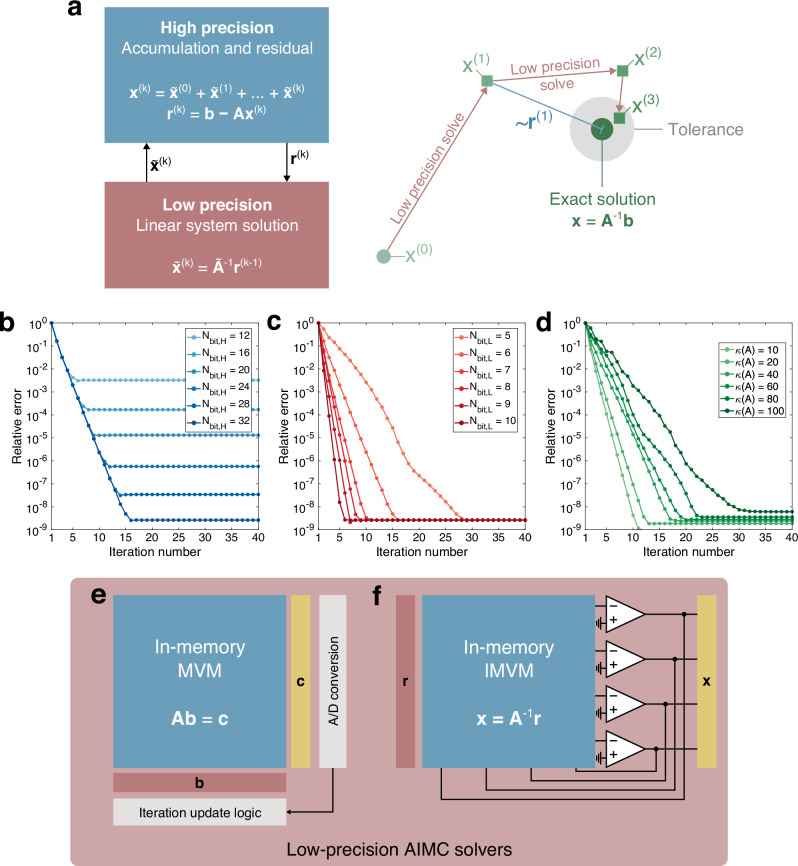
Iterative refinement for linear systems solutions. **a** At each iteration, an efficient low-precision unit computes a correction of the solution ${\tilde{{\bf{x}}}}^{(k)}$ by solving the linear system associated with the residual which is, instead, computed by a high-precision unit. The algorithm takes advantage of the high-precision unit to increase the precision of the numerical solution, whereas the demanding computation is delegated to the low-precision unit. The process stops when the target tolerance is achieved on the residual value. **b**–**d** Relative error as a function of the iteration number for 50 × 50 linear systems for (**b**) 6-bit linear system solver and increasing high-precision residual computation from 12-bit to 32-bit (*κ* = 50), (**c**) 32-bit residual computation and increasing low-precision solver from 5-bit to 10-bit (*κ* = 50), and (**d**) 6-bit linear system solver, 32-bit residual computation unit and increasing linear system condition number from *κ* = 10 to *κ* = 100. **e**–**f** Possible implementations of the LPU by (**e**) iterative open-loop AIMC-based MVM, or (**f**) one-shot closed-loop AIMC-based IMVM.

IRF is based on the iterative computation of a sequence of candidate solutions {**x**^(*k*)^} which converges to the exact solution **x** of the linear system **Ax** = **b**. Figure 6a conceptually illustrates the workflow of IRF. Starting from a solution candidate **x**^(*k*−1)^, IRF computes the residual **r**^(*k*)^ as:

15 $${{\bf{r}}}^{(k)}={\bf{b}}-{\bf{A}}{{\bf{x}}}^{(k-1)}.$$

A correction ${\tilde{{\bf{x}}}}^{(k)}$ is then computed by solving a linear system over an inaccurate matrix $\tilde{{\bf{A}}}$ and known term **r**^(*k*)^, i.e.:

16 $${\tilde{{\bf{x}}}}^{(k)}={\tilde{{\bf{A}}}}^{-1}{{\bf{r}}}^{(k)},$$

such that a new solution candidate is obtained as:

17 $${{\bf{x}}}^{(k)}={{\bf{x}}}^{(k-1)}+{\tilde{{\bf{x}}}}^{(k)}=\mathop{\sum }\limits_{i=0}^{k}{\tilde{{\bf{x}}}}^{(i)},$$

Figures 6b–d show the relative solution error as a function of the iteration number for 50 × 50 linear systems, and varying (b) residual computation and (c) inaccurate linear system solution accuracies, and (d) linear system condition number, highlighting an exponential improvement with each iteration. Notably, the IRF solution precision is ultimately limited by the residual computation accuracy *N*_*bit*,*H*_ of Eq. (15) (Fig. 6b), whereas the accuracy of the approximate linear system solution *N*_*bit*,*L*_ typically influences the convergence rate (Fig. 6c), i.e. the overall number of iterations required to meet a desired tolerance. As a consequence, IRF-based systems typically employ units with different precisions to implement Eqs. (15), (16), with high-precision (HP) unit used for residual computation, and low-precision (LP) unit providing linear system solutions with reduced computational complexity at each iteration. Finally, both the convergence rate and ultimate precision are influenced by the linear system condition number *κ* as highlighted in Fig. 6d, both typically exhibiting ${\mathcal{O}}(\kappa )$ complexity^122^.

The possibility of combining the high accuracy of IRF with the high parallelism of AIMC was first proposed by Richter^123^. In^49^, Le Gallo et al. realize the HP unit with a digital floating-point processor, whereas the LP IMVM solver is composed of a CBA-based MVM accelerator iteratively operated under a Krylov subspace linear solver as shown in Fig. 6e, namely conjugate gradient (CG). By combining an inner LP iterative solver based on low-power, highly-parallel CBAs and outer digital-based HP residual computation, an accuracy of 10^−6^ was demonstrated in solving well-conditioned linear systems described by prototype covariance matrices. By implementing the iterative update circuitry for the inner solver at the analog level, Li et al.^124^ further demonstrated up to 10^−12^ solution accuracy for time-evolving partial differential equations. Feinberg et al.^125^ further propose replacing the iterative inner solver with the one-step, IMC-based IMVM solver^25^ shown in Fig. 6f to further increase the solution throughput.

IRF is known to exhibit a slow convergence rate or divergence for ill-conditioned linear systems^126^. Kalantzis et al.^127,128^ proposed a preconditioned Richardson iterator for the outer IRF algorithm, demonstrating improved robustness in the solution of sparse linear systems. Preconditioners may also be embedded directly within the AIMC primitives^41^ to mitigate the impact of ill-conditioning on the low-precision solver. Most recently, Wu et al.^129^ formulated a stable IRF outer algorithm with improved robustness and superior convergence performance with respect to vanilla IRF.

## Discussion

While each of the presented methodologies has been successfully implemented to achieve the results summarized in Table 2, precision improvement in AIMC systems exhibits tradeoffs in terms of complexity, area, and digital overhead. Slicing methods allow the representation of high-bit-precision operands by relatively low-precision memory devices and data converters. Partial results obtained by low-precision computations can be linearly combined in either the digital domain by S&A or in the analog domain by leveraging current integration on capacitive readout systems. Partial operations can be executed in parallel by replicating matrix slices onto multiple cores, thus preserving the throughput advantage of IMC systems. This approach, however, incurs a significant area overhead owing to the replication of both memory arrays and circuit peripherals, such as the high-precision ADCs for output conversion, as well as requiring frequent tile-to-tile data movement for the accumulation of partial results. Area occupation and tile-to-tile communication may be reduced by spatially mapping tiles onto the same memory array, at the cost of either operating on smaller target matrices if the same memory array size is considered, or imposing stricter requirements on array nonidealities such as IR drop and driving capabilities of the DAC peripherals to retain operability on the same matrix size. Intermediate approaches, where multiple physical arrays are used, each storing multiple weight slices, may allow for the optimal balancing of inter-tile communication and tile accuracy constraints.

**Table 2:** Representative works on error mitigation in AIMC systems and quantitative benchmarks

Authors	Year	Technique	Main result
Hu et al.^77^	2016	Slicing	7-bit computation on 4-bit primitive by binary slicing (+3 bits)
Shafiee et al.^76^	2016	Slicing	16-bit computation by binary matrix and input slicing (+15 bits)
Ankit et al.^80^	2019	Slicing	16-bit computation by binary matrix and input slicing (+15 bits)
Pedretti et al.^79^	2021	Slicing	11-bit weight precision on 4-bit memory by analog slicing (+7 bits)
Le Gallo et al.^75^	2022	Slicing	Binary slicing optimization for bit-width and drift mitigation
Khaddam-Aljameh et al.^13^	2022	Slicing	7-bit input slicing by PWM with time-to-digital readout
Jiang et al.^74^	2022	Slicing	Analog input slicing with capacitive readout and PWM generation
Song et al.^21^	2024	Slicing	Arbitrary precision on 3-bit memory by analog slicing (≈ +13 bits)
Salamat et al.^94^	2018	RNS	Simulated 16-bit accuracy on 6-bit analog CBA (+10 bits)
Roohi et al.^95^	2021	RNS	Simulated 16-bit accuracy on 5-bit digital CBA (+11 bits)
Demirkiran et al.^85^	2024	RNS	FP32 inference accuracy on 6-bit analog primitives (≈ +18 bit)
Niu et al.^107^	2012	ECC	Bit-level CBA protection by Hamming code-based ECC
Feinberg et al.^109^	2018	ECC	16-bit compute-level protection by ABN ECC
Li, Roth et al.^66^	2020	ECC	Correction of outlying errors by fully-analog ECC
Li, Read et al.^110^	2022	ECC	In situ analog/digital AN code-based ECC for MSB correction
Parrini et al.^136^	2024	ECC	4-bit weight protection by analog ECC
Le Gallo et al.^49^	2018	IRF	10^−6^ solution accuracy with 4-bit AIMC primitive (+16 bits)
Feinberg et al.^125^	2021	IRF	In situ preconditioning by AIMC IMVM
Kalantzis, Gupta et al.^127^	2021	IRF	AIMC-preconditioned linear system solution by Richardson iteration
Wu et al.^129^	2023	IRF	AIMC-tailored iterative update algorithm
Kalantzis, Squillante et al.^128^	2023	IRF	AIMC-preconditioned linear system solution by GMRES algorithm
Li, Xue et al.^124^	2025	IRF	10^−10^ accuracy with fully-analog iterative AIMC solver

Area occupation can be reduced by adopting RNS-based decomposition, where the required precision of DACs, memory, and ADCs is determined by the integer moduli. Partial operations can be executed in parallel on multiple cores, each performing computations with respect to a modulo of the selected set. However, the digital circuitry must address the modular arithmetic to perform reconstruction by CRT with medium-high complexity. With respect to slicing, RNS displays a higher sensitivity to noise and perturbations, leading to correlation loss between the computed output and the expected output. RNS robustness can be improved by introducing redundant moduli, allowing detection and correction of shot-like noise events, or by selecting non-pairwise-coprime integers as moduli at the cost of increased reconstruction overhead. Noise rejection may be further enhanced by ECCs for the detection and correction of outlying noise events, the identification of highly variable devices, and the restoration of nominal precision.

By suitably combining slicing, RNS, and ECC, high-precision in-memory MVM units can be obtained from low-precision memory and peripherals. High-precision MVM represents a fundamental cornerstone for the iterative solution of linear systems, typically relying on the construction of fixed-point sequences converging to the desired IMVM result. While performing all the MVMs required by the iterative solver in a high-precision unit may result in significant energy consumption, IRF can be used to delegate a significant fraction of the required computations to a lower-precision unit with reduced latency and energy consumption. In this scheme, closed-loop AIMC may play a pivotal role by acting as a one-step, low-precision solver, with the enhanced-precision open-loop AIMC primitive providing high-precision refinement.

Table 3 summarizes the reviewed error mitigation techniques and their impact on computation precision, as well as some qualitative benchmarks in terms of latency, area consumption, and required digital overhead. Slicing, RNS, and IRF allow high-precision computation outputs by suitably combining low-precision partial results. Conversely, ECCs are typically limited to restoration of the nominal primitive precision by improving its robustness to noise and perturbations. Slicing, RNS, and ECC can be implemented with minimal latency by adopting spatial parallelism for slicing and RNS, and MVM-based encoders and decoders for ECC. Conversely, the sequential nature of IRF may result in increased latency for the high-precision computation with respect to the low-precision IMVM solution. The spatial parallelism of RNS and slicing leads to significant area consumption, with RNS typically being advantageous with respect to slicing owing to the reduced bit-precision of ADCs. The area overhead of IRF can be reduced by reusing high-precision in-memory MVM accelerators for residual computation, avoiding the need for digital coprocessors. Implementation of modular arithmetic for reconstruction by CRT leads to a significant digital overhead required for RNS, whereas slicing can exploit S&A with reduced digital complexity. Syndrome computation in ECC may similarly require the implementation of modular arithmetic, although with a vastly reduced number of operands and, thus, a more lightweight circuitry requirement.

**Table 3:** Qualitative benchmarks for error mitigation techniques

Technique	Impact on precision	Latency	Area	Digital overhead	Data movement
Slicing	Increase (MVM)	Low	High	Low	High
RNS	Increase (MVM)	Low	Low	High	Medium
ECC	Recovery (MVM)	Low	Low	Medium	Low
IRF	Increase (IMVM)	High	Medium	Medium	High

While this work focuses primarily on resistive AIMC, similar precision challenges arise in all analog computing contexts based on other physical domains. For instance, Zhou et al. demonstrated a photonic-electronic in-memory dot-product engine with 4-bit weight encoding, mainly limited by photodetector, shot, and thermal noise^130^. On the ferroelectric side, Soliman et al. showed a multi-level FeFET crossbar IMC implementation capable of multi-bit MAC operations, where bit-precision of stored weights is limited by device variability^131^. More recently, dual-design FeFET AIMC architectures with inherent shift-add mechanisms have been proposed to enhance precision and reduce overhead^132^. In quantum computing, decoherence and imperfect gate operations necessitate extensive use of quantum ECCs^133^ and noise mitigation techniques^134,135^. While these works do not yet employ full precision-enhancement techniques, they underscore the cross-platform relevance of error mitigation in in-memory computing and suggest fertile ground for applying our surveyed approaches more broadly.

## Conclusions

This work provides an overview of error mitigation techniques aimed at elevating the computing precision of inherently low-precision AIMC primitives, with a specific focus on MVM and IMVM operations. The main sources of error affecting analog computing primitives are first identified, including memory, DAC, and ADC quantization, variability, and noise, as well as circuit-level nonidealities. Slicing and RNS are introduced to relax the precision requirements for the memory and converters by decomposing high-bit-width inputs and matrices into low-bit-width operands. To reject noise and allow identification of faulty devices, three different ECC implementations are reviewed, namely Hamming and ABN codes, as well as fully-analog, AIMC-tailored ECCs. Finally, IRF is proposed as an IMVM-specific algorithm to allow high-precision solution of matrix equations by leveraging low-precision in-memory linear system solution, either by iterative open-loop AIMC or one-shot closed-loop AIMC. While the techniques reviewed in this work have been individually demonstrated in real AIMC prototypes, we argue that combining different error mitigation and precision improvement strategies within the same AIMC core can yield greater precision gains while offsetting the limitations of each individual approach. A promising direction for future work will be the development of systematic numerical studies, combining different modulation schemes, noise sources, and input distributions, to quantitatively assess the range of achievable precision improvements and trade-offs across techniques. A careful co-integration of these frameworks can enable harnessing the intrinsic energy and area efficiency of AIMC while achieving the high precision typically required by advanced data processing, thereby advancing AIMC systems toward real-world applicability.

## Data Availability

No datasets were generated or analysed during the current study.
